# Salivary Calculi

**Published:** 1871-12

**Authors:** 


*Salivary Calculi*—Dr. J. Wyatt Pratt relates the following interesting case in the "London Lancet."

"L. P——, a retired medical gentleman, aged fifty four; has been lately in good health, but formerly exhibited indications of strumous diathesis. Twenty years ago he suffered from symptoms of phthisis, and coughed up, during much bronchial irritation, several chalky concretions. Has passed, at varying intervals of a few years, without much discomfort, small single calculi from the left submaxillary duct. After three or four years' relief, on the 6th and 7th of July last he commenced to void many small calculi from the same situation, accompanined with painful mastication and uneasiness on the floor of the mouth.

July 8th—Has had a bad night. At 10 A. M. he was found with both parotid, both submaxlllary, and both sublingual glands much inflamed and enlarged; tonsils and uvula much increased in size, rendering deglutition very difficult, all food regurgitating into the posterior nares unless the nose was tightly held during attempts to swallow; tongue considerably enlarged, and covered with creamy fur; power of articulation almost lost; elevation at the termination of the left Whartonion duct prominent, and the duct itself distended by a small calculus, which was removed by Dr. Edwards,—my senior partner—through the natural orifice, with a probe and pair of fine forceps, after which a quantity of saliva escaped and a little bead of pus. Opposite to the left lower first molar was a transparent vesication on the mucous membrane, over the sublingual gland, upon puncturing which a quantity of clear saliva looking fluid escaped. Skin hot; pulse full and bounding, nearly 120; bowels confined; both cheeks puffy and much distended; breath very offensive, and saliva dribbling freely from the mouth. Ordered, two grains of calomel immediately; to inhale steam; brandy and milk diet; and twenty-five minims of chlorodyne at night.

9th—Had a better night, and brought away two or three more calculi. A square block of white, smooth, firm lymph, as large as the crown of a molar tooth, had been secreted from the surface where the vesication had existed, and was now detached as it prodnced discomfort. Duct freely discharging saliva. Pyrexia diminished.

10th.—Restless night. Duct blocked up again: a blunt probe was introduced, and this had the effect of removing a liitle plug of sanious lymph, which was followed by a quantity of offensive pus. No calculi since yesterday Block of lymph on the surface removed as before.

11th.—Continual discharge of healthy-looking pus from affected duct. Papilla at its entrance hard, and as large as a No. 1 shot. Block of lymph again requiring removal. Swelling reduced.

12th.—Swallows better. Left submaxilly gland still enlarged and tender; other glands diminished to natural size. Free discharge of pus. Ordered nitric acid and liberal diet.

Went on improving until the 22nd. when the largest calculus ulcerated its way through the duct, about half an inch from its mouth, and was easily removed with a pair of forceps.

From this time he progressed favorably. The fistulous opening caused by last calculus soon healcd, and the discharge gradually ceased, though the floor of the mouth remained tender and irritable for three or four weeks.

The number of calculi passed during the fortnight was thirteen, varying in size from that of a good-sized pin's head to the largest, which is rather bigger than a full-sized grain of wheat, and was the only one that did not escape from the natural aperture of the gland duct. They are of a pale-fawn color, roundish, slightly rough, and somewhat porous, and of a very light specific gravity.

